# Activation of a Bovine Mammary Epithelial Cell Line by Ruminant-Associated *Staphylococcus aureus* is Lineage Dependent

**DOI:** 10.3390/microorganisms7120688

**Published:** 2019-12-12

**Authors:** Jurriaan Hoekstra, Victor P. M. G. Rutten, Theo J. G. M. Lam, Kok P. M. Van Kessel, Mirlin P. Spaninks, J. Arjan Stegeman, Lindert Benedictus, Gerrit Koop

**Affiliations:** 1 Department of Farm Animal Health, Faculty of Veterinary Medicine, Utrecht University, 3584 CL Utrecht, The Netherlands; t.lam@gddiergezondheid.nl (T.J.G.M.L.); M.P.Spaninks@uu.nl (M.P.S.); J.A.Stegeman@uu.nl (J.A.S.); L.Benedictus1@uu.nl (L.B.); g.koop@uu.nl (G.K.); 2 Department of Infectious Diseases and Immunology, Faculty of Veterinary Medicine, Utrecht University, 3584 CL Utrecht, The Netherlands; v.rutten@uu.nl; 3 Department of Veterinary Tropical Diseases, Faculty of Veterinary Science, University of Pretoria, 0110 Onderstepoort, South Africa; 4 GD Animal Health, 7418 EZ Deventer, The Netherlands; 5 Department of Medical Microbiology, University Medical Center Utrecht, 3584 CX Utrecht, The Netherlands; K.Kessel@umcutrecht.nl

**Keywords:** *Staphylococcus aureus*, clonal complexes, bovine mammary epithelial cells, mastitis, immune response, IL-8

## Abstract

Bovine mastitis is a costly disease to the dairy industry and intramammary infections (IMI) with *Staphylococcus aureus* are a major cause of mastitis. *Staphylococcus aureus* strains responsible for mastitis in cattle predominantly belong to ruminant-associated clonal complexes (CCs). Recognition of pathogens by bovine mammary epithelial cells (bMEC) plays a key role in activation of immune responsiveness during IMI. However, it is still largely unknown to what extent the bMEC response differs according to *S. aureus* CC. The aim of this study was to determine whether ruminant-associated *S. aureus* CCs differentially activate bMEC. For this purpose, the immortalized bMEC line PS was stimulated with *S. aureus* mastitis isolates belonging to four different clonal complexes (CCs; CC133, CC479, CC151 and CC425) and interleukin 8 (IL-8) release was measured as indicator of activation. To validate our bMEC model, we first stimulated PS cells with genetically modified *S. aureus* strains lacking (protein A, wall teichoic acid (WTA) synthesis) or expressing (capsular polysaccharide (CP) type 5 or type 8) factors expected to affect *S. aureus* recognition by bMEC. The absence of functional WTA synthesis increased IL-8 release by bMEC in response to bacterial stimulation compared to wildtype. In addition, bMEC released more IL-8 after stimulation with *S. aureus* expressing CP type 5 compared to CP type 8 or a strain lacking CP expression. Among the *S. aureus* lineages, isolates belonging to CC133 induced a significantly stronger IL-8 release from bMEC than isolates from the other CCs, and the IL-8 response to CC479 was higher compared to CC151 and CC425. Transcription levels of IL-8, tumor necrosis factor alpha (TNFα), serum amyloid A3 (SAA3), Toll-like receptor (TLR)-2 and nuclear factor κB (NF-κB) in bMEC after bacterial stimulation tended to follow a similar pattern as IL-8 release, but there were no significant differences between the CCs. This study demonstrates a differential activation of bMEC by ruminant-associated CCs of *S. aureus*, which may have implications for the severity of mastitis during IMI by *S. aureus* belonging to these lineages.

## 1. Introduction

Mastitis is a major economic problem for the dairy industry and the predominant causes of bovine mastitis are intramammary infections (IMI) with bacteria [1]. The mammary gland has a well-developed innate immune system that responds to incoming pathogens during IMI, and bovine mammary epithelial cells (bMECs) play an important role in activating the early innate immune response [2]. Pattern recognition receptors (PRRs), such as Toll-like receptor (TLR) 1, TLR-2, TLR-6, nucleotide-binding oligomerization domain-like receptors (NLR) 1, NLR-2, expressed by bMEC are activated by pathogen-associated molecular patterns (PAMPs) [3], initiating the activation of intracellular signaling pathways that increase expression and release of chemokines, cytokines, and antimicrobial peptides [2]. Proinflammatory cytokines, such as interleukin 8 (IL-8) and tumor necrosis factor alpha (TNFα), initiate the inflammatory response by recruiting and activating leukocytes [4]. Furthermore, antimicrobial peptides, like serum amyloid A3 (SAA3), β-defensins and cathelicidins, directly attack bacteria in the lumen of the mammary gland [5,6,7].

The innate immune response of the bovine mammary gland to different bacterial species varies, most notably between *Staphylococcus aureus* and *Escherichia coli* [8]. This differential response is dictated by a pathogen-specific activation of bMEC, since challenge of bMEC, both in vitro and in vivo, with *S. aureus* results in a weak transcription response compared to challenge with *E. coli* [9,10]. This might be explained by the large number of immune evasion molecules produced by *S. aureus*, several of which affect host recognition of the bacteria [11]. For instance, the exo-protein Staphylococcal superantigen-like protein (SSL) 3 binds to the extracellular part of TLR-2 without activating the cell [12]. Moreover, *S. aureus* reduces contact between PAMPs and host PRRs by covering its surface with capsular polysaccharides (CPs) [13] and wall teichoic acids (WTAs) [14]. Experimental overexpression or deletion of these immune evasion factors affects the virulence of *S. aureus* [12,14].

*Staphylococcus aureus* responsible for IMI in cattle predominantly belong to ruminant-associated clonal complexes (CCs), such as CC151, CC97, CC479, and CC133 [15,16] and recently, it was reported that the in vitro bMEC response to *S. aureus* differs between some of these CCs [17,18]. Strains belonging to CC151 induce a lower release of proinflammatory cytokines from bMEC than CC97, CC71 and sequence type (ST) 136 *S. aureus* [17,18]. Whereas most CCs used in these in vitro studies were bovine-associated, little is known about CCs of *S. aureus* associated with mastitis in small ruminants, such as CC133, CC130 and CC425 [19,20]. It is known that *S. aureus* can jump species barriers, and cause disease in a new host species [21]. It is important to understand the processes that enable *S. aureus* to switch hosts in order to find new targets to control infections [22]. Small ruminant associated *S. aureus* have been shown to occasionally infect cattle (e.g., [16]), but it is unknown to what extent the immune response to these lineages differs from infections with bovine associated *S. aureus* lineages. Clonal complex 479 has been associated with severe bovine mastitis cases [16,23], hence, the effects of CC479 strains on bMEC, which have not been studied thus far, may yield valuable insights into the pathogenesis of this lineage. Since the strength of proinflammatory responsiveness of bMEC towards invading pathogens is expected to influence the course and outcome of IMI, increased understanding of the interaction between *S. aureus* belonging to different CC and bMEC during IMI can give insight in the variable pathogenicity of *S. aureus* lineages in bovine mastitis.

In the present study, the potential of four *S. aureus* CCs, associated with bovine and small ruminant mastitis, to activate bMEC was investigated. The spontaneously immortalized bMEC line PS was used as a model for bMEC. This cell line expresses the same PRRs as primary bMEC, has a stable cell morphology and responds to artificial TLR-2 ligands Pam2, Pam3 and control PAMPs (lipopolysaccharides, lipoteichoic acid), making it a useful surrogate for primary bMEC [24]. To validate our assay, we used genetically modified *S. aureus* lacking factors (WTA, CP and protein A) expected to affect recognition of *S. aureus* by bMEC.

## 2. Materials and Methods

### 2.1. PS Cell Culture Conditions

The PS cell line, a spontaneously immortalized cell line originating from cultured primary bMEC that was kindly donated by Dr Pascal Rainard and Dr Pierre Germon of the French National Institute for Agricultural Research, was used as a model for bMEC [24]. PS cells were cultured in growth medium (GM) consisting of DMEM/F12 medium (Thermofisher, Waltham, MA, USA) containing 1 μg/mL hydrocortisone (Merck, Kenilworth, NY, USA), 10 ng/mL insulin-like growth factor 1 (IGF-1) (Peprotech, London, UK), 5 ng/mL fibroblast growth factor (FGF) (Peprotech), 5 ng/mL epidermal growth factor (EGF) (Merck), 20 mM HEPES (Merck) and 2 mM Glutamax (Thermofisher).

### 2.2. Bacteriological Culture

A panel of *S. aureus* isolates (*n* = 35) obtained from cases of ruminant mastitis in the Netherlands was used. Isolates were randomly selected from an in-house *S. aureus* collection, previously described by Hoekstra et al. [20,21], to represent the bovine-associated CCs 151 (*n* = 7), 479 (*n* = 9) and small ruminant-associated CCs 133 (*n* = 15) and 425 (*n* = 4). In addition, a set of genetically modified and corresponding wildtype (wt) *S. aureus* strains (Table 1) were selected to validate the PS cell system. Strain RN4220 *ΔtarO* (belonging to CC8) lacks an essential gene for WTA synthesis, resulting in the absence of WTA, and RN 4220 *ΔtarS/ΔtarM* lacks genes required for glycosylation of WTA [25]. These isolates were kindly donated by Prof. Andreas Peschel, University Tubingen, Germany. The wt Reynolds strain (belonging to CC25) carries the CP type 5 (*cap5*) gene, which is replaced by CP type 8 gene (*cap8*) in the genetically modified Reynolds *cap8* strain and deleted in the Reynolds *Δcap5* strain [26]. These isolates were kindly donated by Jean C. Lee, Division of Infectious Diseases, Brigham and Women’s Hospital, Boston. In the Newman *Δspa* strain (belonging to CC8), the gene coding for surface protein A (*spa)* is deleted [27], and these strains were kindly donated by Prof. Jan Maarten van Dijl, University Medical Center Groningen, The Netherlands.

Bacteria, from glycerol stocks, were cultured overnight at 37 °C on blood agar plates. Several colonies were picked and washed in 50 mL PBS. After centrifuge (4000× *g* for 10 min), bacteria were resuspended in 5 mL of stimulation medium (SM), consisting from DMEM/F12 medium (Thermofisher) containing 20 mM HEPES (Merck) and 2 mM Glutamax (Thermofisher). The number of colony forming units (CFU)/mL was estimated using the optical density (OD) at 660 nm, based on the conversion OD660 of 0.5 equals 5 × 10^8^ CFU/mL, which was confirmed by plating and counting bacterial suspensions prior to performing the assays. Bacterial suspensions were diluted further to the appropriate CFU/mL for stimulation experiments.

### 2.3. PS Cell Responses to S. aureus

To study the bMEC responses towards *S. aureus*, we used 10^5^ PS cells at passage number 13, which was the earliest passage we could use to obtain enough cells to perform all experiments. These cells were seeded in a volume of 1 mL per well in a 24-well plate and cultured in GM until they formed an approximate 80% confluent monolayer. They were then cultured overnight in SM. During these periods, the cells formed a monolayer, but did not show growth in number of cells, so we assumed the numbers of cells to have remained 10^5^ per well. After two washing steps with phosphate-buffered saline (PBS) (Merck), PS cell monolayers were exposed to wt or genetically modified *S. aureus* in SM at a multiplicity of infection (MOI) of 160, by adding 1.6 × 10^7^ bacteria. The MOI was based on pilot experiments that demonstrated that at this multiplicity, PS cells have the strongest IL-8 response to *S. aureus* in the absence of cytotoxic effects, as assessed by trypan blue staining and microscopy examination. The same maximum MOI for stimulating PS cells with *S. aureus* was also found by Deplanche et al. [28]. After three hours, bacteria were removed by washing cells two times with PBS, followed by incubation in 0.5 mL SM containing 100 µg/mL streptomycin and 100 I.U./mL penicillin to prevent growth of bacteria. After five hours, the supernatant of each well was collected and stored at −20 °C. Supernatant of unstimulated cells was used as negative control. As a positive control, cells were exposed to 100 ng/mL of the synthetic TLR2 agonist Pam2-CSK4 (InvivoGen, San Diego, CA, USA). For each isolate, the stimulation assay was performed in triplicate.

### 2.4. IL-8 ELISA

IL-8 production by PS cells was measured by ELISA designed using the bovine IL-8 development kit (MabTech, Nacka Strand, Sweden). ELISA 96 wells microplates (Corning Inc., Corning, NY, USA) were coated overnight at 4 °C using 50 µL of 0.5 µg/mL monoclonal antibody MT8H6 diluted in PBS. After a washing step with 300 µL 0.25% Tween20 in PBS, plates were blocked using 200 µL blocking reagent (Roche Diagnostics, Risch-Rotkreuz, Switzerland). Next, 100 µL of supernatant or IL-8 standards were added to 96 well plates and were incubated under agitation at room temperature for 1 h. Following a washing step with 300 µL 0.25% Tween20 in PBS, 50 µL of biotinylated monoclonal antibody 26E5 (0.1 µg/mL diluted in blocking reagent) was added. Following 1 h of incubation at room temperature, the plate was washed again and 100 µL of Streptavidin-HRP (1:2000 dilution in blocking reagent) (BD Biosciences, Franklin Lakes, USA) was added. After 1 h of incubation and a final washing step, 100 µL of HRP substrate 3,3’,5,5’-tetramethylbenzidine (Merck) was added and the reaction was stopped after 15 min using H_2_SO_4_. Extinctions (450 nm) were measured on a Microplate Reader. Using a standard curve, the IL-8 levels in supernatant were extrapolated using GraphPad Prism version 7 (GraphPad Software, La Jolla, CA, USA). The detection range of the bovine IL-8 ELISA was 2–250 pg/mL.

### 2.5. Total RNA Extraction and Reverse Transcription

Transcription levels of TNFα, SAA3, NF-κB and TLR-2 genes of PS in response to randomly selected CC151 (*n* = 4), CC479 (*n* = 4) and CC133 (*n* = 4) isolates were measured using quantitative real-time PCR (qPCR). For these experiments, 2 × 10^5^ cells per well were seeded in a 12-well plate and cultured until 80–90% confluence. Stimulation with bacteria was performed using the same protocol and volumes as described in Section 2.3. Five hours after removal of bacteria, RNA was extracted directly from monolayers within the 12-well plates using the RNeasy Micro Kit (QIAGEN, Venlo, The Netherlands) according to the manufacturer’s instructions and converted to cDNA using the iSCRIPT cDNA Synthesis Kit (BioRad, Hercules, CA, USA). Primers for genes of interest (IL-8, TNFα, SAA3, NF-κB, TLR-2) were taken from literature and are displayed in Table 2. Quantitative real-time PCR (qPCR) Master-mix was prepared as follows: 5 µL Iq Sybr green Master-mix (Biorad), 1 µL of forward and reverse primers each (final concentration 100 nM), 1 µL of demineralized water and 2 µL of 1:20 diluted cDNA. For qPCR, a three-step PCR protocol was used: An initial 10 min denaturation at 95 °C, followed by 40 cycles with 15 s of denaturation at 95 °C, 30 s annealing at primer specific temperature, and 30 s of elongation at 72 °C. Fluorescence was detected after each cycle. At the end of the first PCR the melting curve of the PCR product was determined for each primer set to verify the specificity of the PCR reaction, but not in subsequent PCRs using the same primer set. A 10-fold serial dilution of a positive control sample was run in each reaction to estimate the efficiency of the qPCR and the relative gene transcription was calculated using the comparative *C*t method [26].

### 2.6. Statistical Analysis

All statistical analyses were performed using GraphPad Prism 7 software. Each isolate was tested in triplicate and genetically modified strains and their wt counterpart in quadruplicate. Supernatant of each replicate was measured twice by ELISA. For statistical testing, averaged IL-8 levels in supernatant were log10 transformed and analyzed by ANOVA followed by Tukey’s Multiple comparison (TMC) test. When only two groups were available, the Student’s T test was performed. Correlations between IL-8 protein production levels and IL-8 gene transcription, and between log-transformed IL-8, TNFα, NF-κB and TLR-2 gene transcription were analyzed using Pearson’s correlation coefficient. A significance level of 0.05 was used for all tests.

## 3. Results

### 3.1. WTA and CP, but not Protein A, Modulate Activation of PS Cells

**Figure 1:**
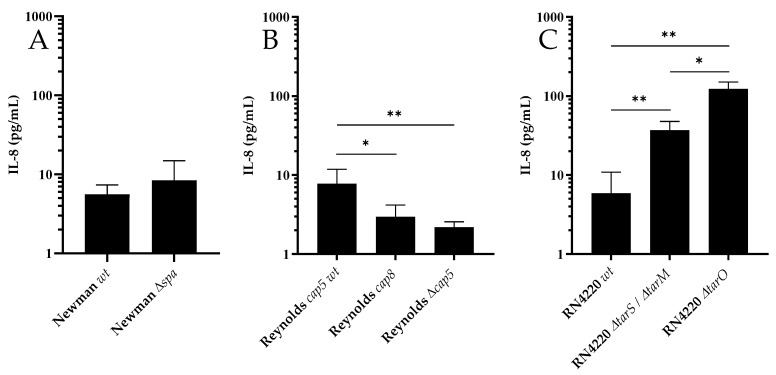
Interleukin 8 (IL-8) production by PS cells after stimulation with wt and genetically modified strains to investigate the effect of protein A (**A**), capsular polysaccharides (**B**) and wall teichoic acid (WTA) (**C**) on bovine mammary epithelial cells (bMEC) activation. Supernatant was collected 5 h after the end of bacterial stimulation. For each bacterial strain, stimulation was performed four times and each supernatant was measured twice by ELISA. Bars show average IL-8 production ± SD. Levels of IL-8 were log10 transformed and analyzed using one-way ANOVA followed by Tukey’s multiple comparisons test or the Student’s T test (** *p* < 0.01, * *p* < 0.05).

PS cells released IL-8 (37.7 ± 14.9 pg/mL; *n* = 8) in response to incubation with the synthetic TLR-2 agonist Pam2. The wt strains RN4220 (5.9 ± 4.9 pg/mL), Newman (5.6 ± 1.8 pg/mL) and Reynolds *cap5* (8.0 ± 4.0 pg/mL) induced a comparable IL-8 release from PS cells (Figure 1). The IL-8 release from PS cells after stimulation with Newman *Δspa* strain (8.4 ± 6.4 pg/mL) and its wt counterpart (Figure 1A) did not differ. Stimulation with the genetically modified Reynolds *cap8* (3.0 ± 1.2 pg/mL) and Reynolds *Δcap*5 (2.2 ± 0.4 pg/mL) induced weaker IL-8 releases (Figure 1B) compared to Reynolds *cap5* wt (ANOVA; *p* = 0.004). Stimulation with the WTA deficient RN4220 *ΔtarO* (122.6 ± 27.3 pg/mL) and RN 4220 *tarS*/*tarM* (36.8 ± 10.7 pg/mL) strains resulted in higher IL-8 production by PS cells than the RN4220 wt (ANOVA; *p* < 0.001, Figure 1C).

### 3.2. IL-8 Production by PS Cells Stimulated with S. aureus Isolates

**Figure 2:**
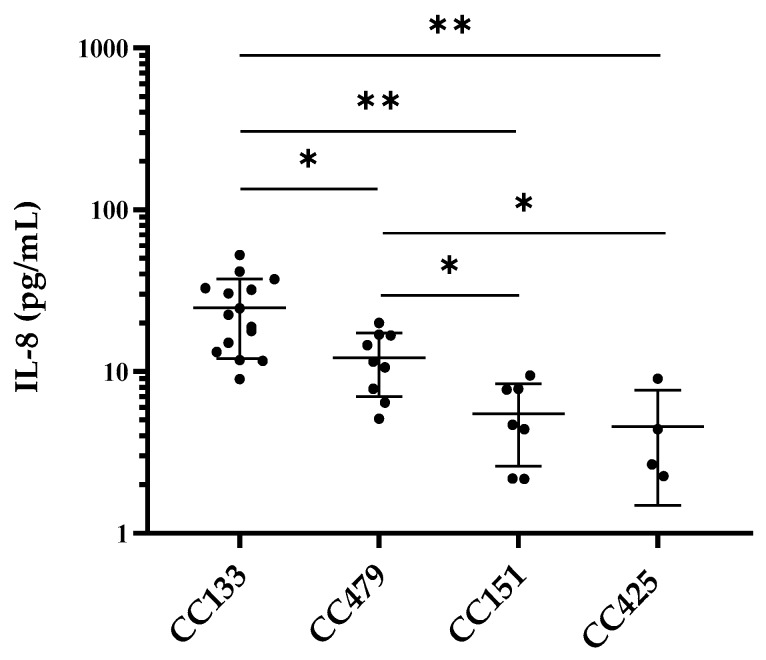
IL-8 production by PS cells in response to stimulation with ruminant-associated *S. aureus* isolates, belonging to CC133 (*n* = 15), CC479 (*n* = 9), CC151 (*n* = 7) and CC425 (*n* = 4). Each dot provides average production of three culture replicates. Each replicate was measured twice by ELISA. Bars show average IL-8 production ± SD. Levels of IL-8 were log10 transformed and analyzed using one-way ANOVA followed by Tukey’s multiple comparisons test (** *p* < 0.01, * *p* < 0.05).

Significant differences in IL-8 release were observed after stimulation of PS cells with *S. aureus* isolates belonging to CC133 (*n* = 15), CC479 (*n* = 9), CC151 (*n* = 7) and CC425 (*n* = 4) (ANOVA; *p* <0.0001). CC133 isolates induced a stronger IL-8 release (24.7 ± 12.7 pg/mL) than CC479 (12.2 ± 5.1 pg/mL), CC151 (5.5 ± 2.9 pg/mL) and CC425 (4.6 ± 3.1 mL) (Figure 2). Furthermore, the release of IL-8 was higher after stimulation of PS cells with CC479 compared to CC151 and CC425.

### 3.3. Transcription of Genes by PS Cells Following Stimulation with S. aureus Isolates

**Figure 3:**
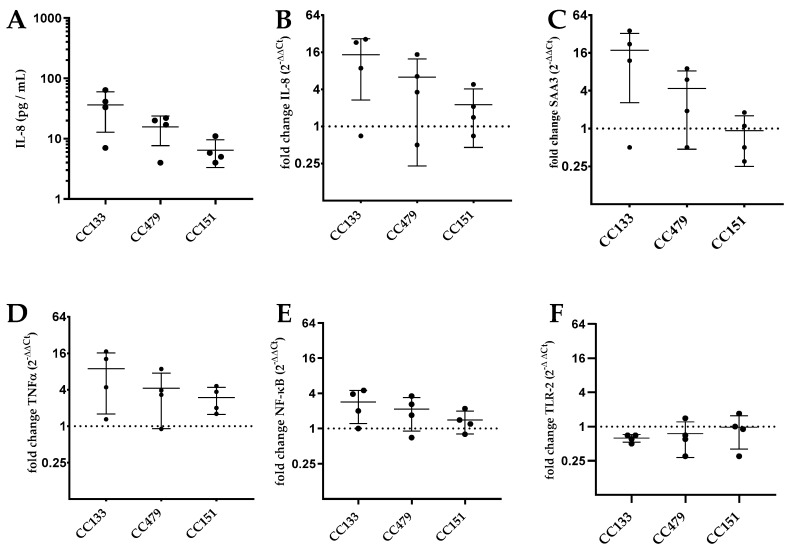
IL-8 production (**A**) and relative transcription of IL-8 (**B**), serum amyloid A3 (SAA3) (**C**), tumor necrosis factor alpha (TNFα) (**D**)*,* NF-κB (**E**) and Toll-like receptor (TLR)-2 (**F**) after stimulation of PS cells with isolates belonging to CC133 (*n* = 4), CC479 (*n* = 4) and CC151 (*n* = 4). For each isolate, the assay was performed in triplicate. IL-8 protein was measured in duplicate by ELISA and transcription of genes by quantitative real-time PCR (qPCR) in triplicate. Relative transcription (2^−ΔΔ*C*t^) was normalized to negative controls and corrected for the reference gene ubiquitin. Log10 transformed IL-8 concentrations and relative transcription were analyzed using one-way ANOVA.

IL-8 production was used as the primary read out parameter for bMEC activation. To further investigate whether IL-8 production after activation of PS cells by *S. aureus* is representative for bMEC activation and the ensuing proinflammatory response in general, the gene transcription levels of IL-8 and TNFα, SAA3, NF-κB and TLR-2 genes were measured after exposure to 12 *S. aureus* isolates as representatives of the three CCs most relevant in ruminants (CC133, CC479, CC151). The association between mRNA transcription levels and IL-8 protein release was quantified. Indeed, release of IL-8 was significantly correlated with relative transcription of IL-8 (Pearson *r* = 0.81; *p* = 0.001), SAA3 (*r* = 0.86; *p* < 0.001) and NF-κB (*r* = 0.78; *p* = 0.02). Furthermore, the transcription of NF-κB was correlated with the transcription of IL-8 (*r* = 0.83; *p* < 0.001) and SAA3 (*r* = 0.82; *p* = 0.001). The release of IL-8 protein by PS cells in response to CC133 (36.3 ± 23.5 pg/mL) was slightly higher, although not significantly, than CC151 (6.4 ± 3.2 pg/mL) (ANOVA; *p* = 0.055) (Figure 3A). Relative transcription levels were not significantly different between PS cells stimulated with isolates of different CCs for IL-8 (ANOVA; *p* = 0.34), TNFα (ANOVA; *p* = 0.46), SAA3 (ANOVA; *p* = 0.10), NF-κB (ANOVA; *p* = 0.41) and TLR-2 (ANOVA; *p* = 0.75) (Figure 3B–F).

## 4. Discussion

During IMI, bMEC are amongst the first cells to come in contact with invading microorganisms. They are crucial for activation of the early innate immune response of the mammary gland and upon recognition of pathogens, bMEC upregulate the expression and release of proinflammatory cytokines [2]. We investigated the potency of four *S. aureus* CCs, commonly associated with ruminant mastitis, to activate the bMEC cell line PS. Using wt and genetically modified *S. aureus* strains, we first validated that our bMEC model could detect variation in *S. aureus* PAMPs. The bMEC activation by *S. aureus* belonging to CC133, CC479, CC151 and CC425 was investigated and there were significant differences in PS cell response between *S. aureus* CCs, most notably CC133 inducing stronger IL-8 release than other CCs.

The first step in the activation of bMEC during IMI is detection of bacterial PAMPs by PRRs [2]. The immortalized bMEC line PS expresses TLR-1,2,6 and NLR-1,2 [24], which recognize Staphylococcal PAMPs, such as peptidoglycan, lipoteichoic acid, lipoproteins and phenol-soluble modulins (PSMs) [3]. As expected, stimulation with the synthetic TLR-2 agonist Pam2 triggered IL-8 release by PS cells. Since intracellular signaling pathways that are activated by PRRs (mitogen-activated protein kinase (MAPK), NF-κB pathway) increase expression of multiple proinflammatory cytokines [32], we used IL-8 production as a proxy reflecting general activation of PS cells.

Using genetically modified *S. aureus* strains, we found that absence of WTA synthesis (RN4220 *ΔtarO*) and glycosylation (RN4220 *ΔtarS/ΔtarM*) in *S. aureus* increases IL-8 release after exposure of PS cells to the pathogen. Similar to peptidoglycan and lipoteichoic acid, WTA is part of the *S. aureus* cell wall but it does not trigger a proinflammatory response from monocytes, and likely also not from bMEC [33]. A possible explanation for increased bMEC activation in response to WTA deficient strains is that the lack of WTA in the outer layer of the cell wall makes the underlying cell wall components more easily accessible for recognition by PRRs. The Reynolds *cap5* wt strain triggered a higher IL-8 response from PS cells than the Reynolds *cap8* or Δ*cap5* strains. Binding of both Staphylococcal CP5 and CP8 to MEC triggers IL-8 release [34], which could explain the decreased IL-8 response to Δ*cap5* compared to *cap5* wt. However, this does not explain the differential response of PS cells to *cap5* and *cap8* strains. Previous work demonstrated that the Reynolds *cap5* strain is more resistant to neutrophil killing than the Reynolds *cap8* strain [26], suggesting differences in host recognition of CP type 5 and CP type 8. We did not find an effect of deletion of *spa* in *S. aureus* on PS cell activation. In an assay employing HEK293 cells transfected with TLR2, Hilmi et al. [35] reported a reduced TLR2 activity after challenge with a spa deletion mutant compared to the wildtype strain. However, they also report that factors other than spa also control the TLR2 activity. Since the decrease in IL-8 release in this study was relatively small and many factors can affect bMEC activation [36], it is possible that our PS assay was not sensitive enough to detect an effect of *spa* deletion on bMEC activation. Alternatively, protein A may not affect PRR recognition in bMEC. Overall, our experiments using genetically modified strains demonstrated that the PS cell assay is capable of detecting differences in PAMP expression and/or factors that mask PAMPs, and therefore is a suitable model to investigate bMEC activation by ruminant-associated *S. aureus* lineages.

Stimulation with CC133 isolates resulted in a higher IL-8 release than all other CCs tested and the IL-8 release triggered by CC479 isolates was higher than that by CC151 or CC425 isolates. Of these selected CCs, only bMEC activation by *S. aureus* belonging to CC151 has been subject of previous studies [17,18]. Similar to our results, CC151 induced a weak cytokine response by both primary bMEC [17] and the immortalized MEC line MAC-T [18] compared to CCs included in those studies, CC71, CC97 and ST136. Variation in bMEC responsiveness to *S. aureus* may depend on either the host MEC or on genetic differences between bacteria. Therefore, we used the bMEC model PS to quantify the bMEC response induced by *S. aureus*. Thus, the observed differential bMEC response depends on variation in gene carriage and/or expression levels between tested *S. aureus* lineages. Although the presence or absence of several genes can affect bMEC recognition, the exact mechanisms for the differential activation of bMEC by *S. aureus* CCs are unknown. Clearly, our experiments showed that the absence of WTA in the bacterial cell wall of *S. aureus* increases bMEC activation and that bMEC responds differently to CP type 5 and CP type 8. The presence of these factors alone cannot explain the differential bMEC response towards CCs, since WTA is present in all *S. aureus* [37] and CCs tested in this study all have CP type 8 [19]. Expression levels of CP and WTA in *S. aureus* is highly variable and are tightly controlled by multiple systems, including repressor of toxins (*rot*) and the accessory gene regulator (*agr*) system, which is divided into four separate groups (I-IV) based on polymorphisms of *agr* genes [37]. In addition, these regulatory systems also control expression and release of PAMPs, such as PSMs [38], and immune evasion factors that inhibit TLR-2 activation [12]. Since the CCs tested in this study belong to different *agr* types (CC133 to *agr* type I and CC151, CC479, CC425 to type II) [16,39] and CC479 *S. aureus* have been reported to carry a non-functional copy of the *rot* gene [23], differences in expression of these *agr*/*rot* controlled genes are likely and could be a possible explanation for the differential bMEC response towards ruminant-associated CCs. In addition, a previous study associated reduced bMEC activation by *S. aureus* with a negative Staphaurex latex agglutination test (SLAT) phenotype [29]. Our results agree with this observation, since CC151 is SLAT negative and CC133 and CC479 are SLAT positive *S. aureus* [19,40]. The SLAT is based on functionality of bacterial surface proteins that mediate adherence to host cells [40], but it is unclear if this is also related to bMEC activation.

This study focusses on differences between *S. aureus* CCs rather than differences in the host that can affect the bMEC response but, nevertheless, it is likely that host factors also affect the recognition of *S. aureus* by bMEC. Single nucleotide polymorphisms in the PRRs TLR-1 and 2 have been associated with increased risk of mastitis in cattle [41], and it is expected that SNPs in TLRs affect the recognition of pathogens. Therefore, it is a possibility that individual animals respond differently to the same bacterial strain of *S. aureus* and thus, results from this study should be confirmed in different bMEC models. Besides variation between individual animals, variation in MEC response to pathogens between different ruminant species is also expected, since TLR-2 receptors of cattle and goats share 92.5% amino acid identity, and between 85.3–95.3% for other TLR (1–10) receptors [42]. We expected that bovine CCs would be more adapted to bovine PPRs, resulting in less activation of bMEC by *S. aureus* belonging to these CCs compared to *S. aureus* of small ruminant-associated CCs. Indeed, CC133 stimulation resulted in a stronger IL-8 release than the bovine-associated CCs, but surprisingly, CC425 induced only a weak response of PS cells. Therefore, we cannot conclude that small ruminant-associated *S. aureus*, in general, induces a stronger immune response in bMEC than bovine associated lineages. In a follow-up study, we will compare the response of bovine MEC and caprine MEC to small ruminant and bovine-associated CCs of *S. aureus* to investigate the effect of the interaction between host species and *S. aureus* lineage on MEC activation.

Release of Il-8 by PS cells was used as a proxy for bMEC activation in this study, and indeed there was a correlation between IL-8 protein release and transcription levels of IL-8, TNFα and SAA3, genes that are all regulated by the NF-κB pathway [2]. However, there were no significant differences in gene transcription levels of PS cells stimulated with a subset of CC133, CC479 and CC151 isolates. This could be attributed to the relatively small number of experiments in which expression could be measured. Due to the strong link between IL-8 protein release and gene expression, we consider the differential bMEC activation by ruminant-associated CCs from our IL-8 protein-based assay as representative of the activation of bMEC.

Since bMEC activation during IMI plays a key role in initiating the immune response of the mammary gland, the level of bMEC activation by pathogens is expected to be of major influence on the course of mastitis [8]. Infections with *E. coli* trigger a strong proinflammatory response in bMEC and often result in more severe cases of mastitis than infections with *S. aureus* [8], and therefore we hypothesize that increased bMEC activation may also increase the severity of IMI by *S. aureus*. Elevated bMEC activity results in a stronger innate immune response, recruiting a higher number of neutrophils to the site of infection and increasing the clearance rate of bacteria from the mammary gland [40]. Although reduced bacterial survival of *S. aureus* can be considered positive for the host, the high influx of neutrophils can also potentially harm the mammary gland due to released reactive oxygen metabolites, matrix metalloproteinase and prolonged periods of diapedesis of leukocytes through mammary parenchymal tissue [43]. When the induced immune response is not effective in clearing the pathogen, the duration of the mastitis will increase and influx of leukocytes will continue, resulting in more tissue damage, and potentially a more severe mastitis. Although the level of bMEC activation by *S. aureus* likely contributes to the severity of an IMI, it is important to emphasize that the clinical severity of IMI depends on several factors, such host conditions and production of bacterial virulence factors [44]. Recently, we observed that *S. aureus* belonging to CC479 are associated with clinical rather than subclinical mastitis cases in cattle [23]. In the current study, CC479 isolates induced a stronger reaction from bMEC than the other bovine-associated lineage CC151, which could potentially contribute to the severity of mastitis. Future in vivo studies are needed to identify the contribution of bMEC activation on the pathogenesis of Staphylococcal mastitis.

In conclusion, the current study investigated bMEC activation by *S. aureus* isolates belonging to four ruminant mastitis-associated CCs (133, 479, 151 and 425) and showed that these CCs differ in their ability to activate bMEC, which may influence the outcome of IMI. Future studies are required to elucidate how the in vitro activation of MEC translates to the in vivo immune response of the mammary gland and how this affects the pathogenesis and clinical outcome of a *S. aureus* IMI.

## Figures and Tables

**Table 1:** List of genetically modified *S. aureus* strains and their corresponding wildtype (wt) used in this study.

Strain Name	Clonal Complex	Relevant Characteristics	Reference
Reynolds *cap5*	25	wt Reynolds strain, expressing *cap5*	[26]
Reynolds *cap8*	25	Substitution of *cap5* region with the *cap8* region	[26]
Reynolds Δ*cap5*	25	Deletion of *cap5*, capsular polysaccharide negative strain	[26]
Newman wt	8	wt Newman strain	[27]
Newman Δ*spa*	8	Deletion of *spa* gene	[27]
RN4220 wt	8	wt RN4220 strain	[25]
RN 4220 Δ*tarO*	8	Deletion of tarO, essential for wall teichoic acid (WTA) synthesis	[25]
RN 4220 Δ*tarS*/Δ*tarM*	8	Deletion of *tarS* and *tarM*, responsible for WTA glycosylation	[25]

**Table 2:** Sequences, product size and annealing temperature of primers for genes representing activation of bMEC.

Gene	Primer Sequence	Product Size (bp)	Annealing Temperature (°C)	Reference
*IL-8*	Fwd: ATGACTTCCAAGCTGGCTGTTG Rev: TTGATAAATTTGGGGTGGAAAG	149	60	[29]
*TNF-α*	Fwd: CCACGTTGTAGCCGACATC Rev: CCCTGAAGAGGACCTGTGAG	155	60	[29]
*SAA3*	Fwd: CTTTCCACGGGCATCATTTT Rev: CTTCGGGCAGCGTCATAGTT	188	60	[30]
*NF-κB*	Fwd: CTGGAAGCACGAATGACAGA Rev: GCTGTAAACATGAGCCGTACC	179	60	[31]
*TLR-2*	Fwd: CATTCCCTGGCAAGTGGATTATC Rev: GGAATGGCCTTCTTGTCAATGG	201	62	[29]
*Ubiquitin*	Fwd: AGATCCAGGATAAGGAAGGCAT Rev: GCTCCACCTCCAGGGTGAT	198	62	[29]
